# Improving Genomic Prediction in Cassava Field Experiments by Accounting for Interplot Competition

**DOI:** 10.1534/g3.117.300354

**Published:** 2018-01-22

**Authors:** Ani A. Elias, Ismail Rabbi, Peter Kulakow, Jean-Luc Jannink

**Affiliations:** * Department of Plant Breeding and Genetics, Cornell University, Ithaca, New York 14853; † International Institute of Tropical Agriculture, Ibadan 200001, Nigeria and; ‡ United States Department of Agriculture-Agricultural Research Station (USDA-ARS), Robert W. Holley Center for Agriculture and Health, Ithaca, New York 14853-2901

**Keywords:** cassava, genomic selection, interplot competition, predictability, GenPred, Shared Data Resources, Genomic Selection

## Abstract

Plants competing for available resources is an unavoidable phenomenon in a field. We conducted studies in cassava (*Manihot esculenta* Crantz) in order to understand the pattern of this competition. Taking into account the competitive ability of genotypes while selecting parents for breeding advancement or commercialization can be very useful. We assumed that competition could occur at two levels: (i) the genotypic level, which we call interclonal, and (ii) the plot level irrespective of the type of genotype, which we call interplot competition or competition error. Modification in incidence matrices was applied in order to relate neighboring genotype/plot to the performance of a target genotype/plot with respect to its competitive ability. This was added into a genomic selection (GS) model to simultaneously predict the direct and competitive ability of a genotype. Predictability of the models was tested through a 10-fold cross-validation method repeated five times. The best model was chosen as the one with the lowest prediction root mean squared error (pRMSE) compared to that of the base model having no competitive component. Results from our real data studies indicated that <10% increase in accuracy was achieved with GS-interclonal competition model, but this value reached up to 25% with a GS-competition error model. We also found that the competitive influence of a cassava clone is not just limited to the adjacent neighbors but spreads beyond them. Through simulations, we found that a 26% increase of accuracy in estimating trait genotypic effect can be achieved even in the presence of high competitive variance.

The main goals of field evaluation are prediction of genetic values for targeted genotypes and selection of elite genotypes for advancement and commercialization. In field trials, above and below ground interplot competition can be expected for resources such as water, light, nutrients, and space. It is important to account for competition when present, as ignoring it can result in increased error in genetic value estimates that can subsequently limit the advancement in the breeding program (Besag and Kempton 1986; Stringer *et al.* 2011; Rebetzke *et al.* 2014). This interplot competition can be confirmed by negative correlation between harvest yield of neighboring plots. The intensity of this correlation depends on the genetic relatedness and spatial arrangement of genotypes in the field (Hinson and Hanson 1962). Bias due to competition is expected to decrease when related genotypes are placed in proximity. However, field trials are often limited to a single plot of a genotype when resources are limited and a large number of genotypes are required to be evaluated. In this scenario, the genetic relatedness can be accounted by incorporating a relationship matrix in the mixed effect statistical model used for prediction (Muir 2005). Genomic selection (GS) models that use the whole genome marker data information in calculating this additive matrix are useful in this scenario. This approach can potentially capture all the quantitative trait loci (QTL) that contribute to the variation in a trait (Hayes *et al.* 2009), and, consequently, can deliver more accurate prediction (Jannink *et al.* 2010). In this study, GS models are extended to quantify potential genotypic and error variation in competitive ability, and, thereby, to improve prediction of genetic values.

Competition is more important in scenarios where genotypes are placed in small plots without bordering. In such plots, performance is more strongly influenced by the competitive ability of neighboring plots. For example, when there is a significant height difference, the yields of shorter genotypes are often depressed by shading from the taller ones (Kempton and Lockwood 1984), which are aggressive in using resources and thus grow fast. Related genotypes when placed together can also exhibit significant competition in a high density field (Kempton 1982).

From an agronomic perspective, there are two popular analytical approaches in estimating competition: the phenotypic interference model (Kempton 1982), where competition is assumed to be directly related to yield from neighboring plots; and the genotypic inference model (Pearce 1957), where competition is assumed to be related to other agronomical characteristics and is therefore a trait of the genotype as a whole. Assuming that competition is directly related to the yield of neighboring individuals is well applicable to root/tuber crops (Besag and Kempton 1986). Studies done by Connolly *et al.* (1993) in potato revealed that accounting for competition in estimation increased the shrinkage of extreme values of yield. This shrinkage indicated that accounting for the competition effect reduced the error in genetic values due to the fact that high yielding genotypes’ performance was at the cost of low yielding/weak genotypes.

In field experiments, error in prediction of genotypic values can also occur due to the presence of spatial heterogeneity. There are ways to incorporate both spatial and competition effects in the same mixed effect model. Spatial correlation can be modeled as a random effect and competition can be modeled by an effect given to the neighboring plots (Durban *et al.* 2001; Matassa 2003). To accommodate both effects Magnussen (1994) suggested modeling them by means of nearest neighbor adjustment and a standard two-way ANOVA in forest trees. Durban *et al.* (2001) used the inference modeling (Besag and Kempton 1986) in order to adjust for competition, and a one-dimensional smoothing spline to account for spatial heterogeneity in sugar beet experiments, but considered direct genetic effects as fixed. Stringer *et al.* (2011) proposed using a first-order autoregressive (AR1) covariance structure (Gilmour *et al.* 1997) for spatial heterogeneity, and second or third order autoregressive residual structure for competition in sugarcane. They also considered genetic effects as random facilitating genotype selection. Pedigree information was used for assessing direct and competition effects by Hunt *et al.* (2013). Their competition modeling was based on the inference model suggested by Besag and Kempton (1986), and its modification suggested by Draper and Guttman (1980). The spatial heterogeneity was controlled using first order AR1 covariance structure (Gilmour *et al.* 1997). Earlier, de Resende *et al.* (2005) used a very similar approach but without considering the genetic relationship. Cappa *et al.* (2015) also suggested (in order to take advantage of the genetic relatedness among individuals) using the additive relationship matrix to estimate direct and competition effects, and a two-dimensional smoothing spline to assess the spatial heterogeneity. In their model, the variance components were estimated using a Bayesian approach. The competition modeling in above-mentioned models was done by modifying the incidence matrix to give weight to the nearest neighbors around a targeted plot Cappa *et al.* (2015). Cappa and Cantet (2008) considered a function of the inverse of distance between the targeted plot and neighbors, as well as the number of neighbors, while designing the competition incidence matrix. The distance function to modify competition incidence matrix was initially suggested by Muir (2005) as a function of the square of the inverse of distance. The models proposing simultaneous fitting of competition and spatial heterogeneity were useful when strong effects of these factors were present in order to accurately estimate the genetic variance (Cappa *et al.* 2016).

In collaboration with the International Institute of Tropical Agriculture (IITA), we analyzed the competitive ability in cassava (*Manihot esculenta* Crantz), a root crop, in order to enhance the selection process in the breeding program. Cassava is a subsistence crop in sub-Saharan Africa, and is the main source of calories for half a billion people (FAO 2004). Breeding in cassava is initiated with the production of seeds. The plant produced from a seed is later used for clonal propagation through stem cuttings. For clonal evaluation, these cuttings are planted in single row plots without border rows. In such a scenario, a differential effect of competition among neighbors that affects the phenotype is a concern among breeders. We propose three different functions of distance to account for interplot competition and reduce the error in predicting genetic value. Unlike in previous studies, we assumed that the competition could go beyond the nearest neighbor but that it declines with distance. We also account for the dimension of plots as competition between neighbors along the longer plot edge would be expected to be higher than that along the shorter edge of a nonsquare plot. Previously, we conducted an exploratory analysis to estimate spatial correlation patterns in the performance of clones in the field (Elias *et al.* 2018). The GS-competition model proposed in this manuscript was also extended to account for the presence of significant spatial correlation.

## Materials and Methods

### Materials and design of experiment

We used information from cassava breeding field trials conducted in 2013 and 2014 by IITA in Ibadan, Ikenne, and Mokwa in Nigeria. The clones used for the trials consisted of IITA Genetic Gain (GG) population in a preliminary yield trial (PYT), population from cycle 1 (C1; progeny of GG), and cycle 2 (C2; progeny primarily of C1). In summary, 83 parents from GG population gave rise to 2187 progenies for C1. Later, 84 C1 and 13 GG clones (total 97) were selected as parents giving rise to 2466 progenies for C2. The clones were assigned to a field in a randomized design using replicated check clones (1–10 check clones). The fields were partitioned into Ranges and Columns, with unreplicated test clones belonging to the same family assigned to adjacent rectangular plots in a Range in C1 and C2 trials. The relatedness of genotypes was calculated based on the additive relationship matrix using all the SNP markers with >1% minor allele frequency. A detailed description of the population used for this study, experimental design, field and plot orientation, plot dimension, and method for calculating the relatedness of genotypes studied can be found in a companion paper (Elias *et al.* 2018). Total number of observations and unique genotypes for each trial and trait can be found in Table 1 and Supplemental Material, Table S5 in File S1. An observation was calculated as the average of all observations in a plot. Four agronomic traits were evaluated to estimate genetic values of clones: fresh weight yield of storage roots (abbreviated here as FYLD), root dry matter content (DM), fresh weight of shoots (SHTWT), and harvest index (HI). The DM is the percentage dry root for 100 g of fresh root. The FYLD is the fresh root weight measured in kilograms. The SHTWT is the total fresh weight of harvested foliage and stems measured in kilograms. The HI is the proportion FYLD to the total harvested weight (FYLD + SHTWT) (Ly *et al.* 2013). The HI indicates the ability of plants to partition biomass to below ground roots that are harvested. The traits FYLD and SHTWT can be considered direct measures of production.

**Table 1:** Root DM, root FYLD and SHTWT at harvest, and HI from various experiments that showed decrease in pRMSE value in Model 1 or 2* compared to Base, and where chi-square values are nonzero

Data	Trait	Model	Decrease in pRMSE(%)	h2 (Base/Model)	Competition Form	*χ*-square
Ibadan_2013_C1	DM (511& 488)	Base/Model1	<1e−3	0.27/0.28	NN (along the long edge only)	1.44 (0.229)
	FYLD (646 & 617)	Base/Model1	1	0.68/0.68	Slow decay (Dist = 5 m; *b* = 0.001)	9.31 (0.002)**
	HI (648 & 628)	Base/Model1	<1e−3	0.44/0.46	Fast decay (*k* = 0.4)	8.23 (0.004)**
	SHTWT (660 & 631)	Base/Model1	1.7	0.57/0.59	Slow decay (Dist = 5 m; *b* = 0.001)	15.52 (8.10E−05)**
Ibadan_2014_PYT	FYLD (152 & 81)	Base/Model1	0.6	0.63/0.64	Slow decay (Dist = 10 m; *b* = 0.001)	2.49 (0.114)
	HI (154 & 81)	Base/Model1	<1e−3	0.46/0.40	Slow decay (Dist = 10 m; *b* = 0.001)	3.17 (0.075)**
	*SHTWT(151 & 81)	Base/Model2	4.5	0.77/0.67	Competition error	5.93 (0.01)**
Ibadan_2014_C1	HI (282 & 265)	Base/Model1	<1e−3	0.46/0.46	NN (all neighbors)	9.47 (0.002)**
Ikenne_2013_C1	DM (627 & 611)	Base/Model1	<1e−3	0.32/0.33	Fast decay (∝ 1/Dist)	3.24 (0.072)**
	SHTWT (781 & 753)	Base/Model1	0.2	0.65/0.64	Slow decay (Dist = 5 m; *b* = 0.001)	4.95 (0.026)**
Ikenne_2014_C1	DM (313 & 284)	Base/Model1	0.94	0.44/44	NN (along long edge only)	7.31 (0.006)**
Ikenne_2014_C2	SHTWT (367 & 342)	Base/Model1	0.13	0.58/0.58	Fast decay (∝ 1/Dist)	1.59 (0.207)
Mokwa_2013_C1	DM (571 & 537)	Base/Model1	0.73	0.37/0.37	NN (all neighbors)	0.29 (0.588)
	FYLD (734 & 694)	Base/Model1	0.43	0.84/0.84	Slow decay (Dist = 5 m; *b* = 0.1)	3.69 (0.055)**
	HI (744 & 701)	Base/Model1	<1e−3	0.61/0.63	Fast decay (*k* =1)	4.38 (0.036)**
Mokwa_2014_C1	DM (287 & 264)	Base/Model1	<1e−3	0.31/0.31	Fast decay (∝ 1/Dist)	1.6 (0.205)
	*FYLD (321 & 296)	Base/Model2	1.73	0.62/0.65	Competition error	6.21 (0.01)**

Number of observations and unique genotypes available for analysis are given in brackets in the column Trait. Narrow sense heritability (h2) of direct genotypic effect is given. Competition form is given bracketed with the maximum distance (Dist) covered and/or parameter values. Chi-square statistic, calculated from the log likelihood values of the Base and Model 1 or 2*, is given with p-value (in brackets). Table S5 in File S1 is an elaborated version of this table.

** Significant values at *α* = 0.1.

### Competition model framework

In a GS model framework, the phenotype of a plot is affected by the genotypic effect for the trait of the clone in the plot. This effect can be estimated using the genomic relationship matrix as follows:

$$\begin{array}{l} \;\;\;Y_{n\times 1}=\mu +Z1_{n\times g}g_{g\times 1}+\epsilon \;\;\;\;\;\mathrm{Base}\\ g∼N\left(0,K_{g\times g}\sigma_{g}^{2}\right)\;\;\;\epsilon ∼N\left(0,I_{n\times n}\sigma_{e}^{2}\right) \end{array}$$

where **Y** is the response variable (*e.g.*, DM); μ is the general mean; **Z1** is the design matrix for genotypic effect, *n* is the number of observations, and *g* is the number of unique genotypes in the data; ***g*** is the vector of genotypic effect; ϵ is the vector of residual error; **I** is the incidence matrix for residual error; and **K** is the genomic relationship matrix (here, the additive relationship matrix).

However, in breeding evaluation trials, the phenotype of a plot is affected both by the genotypic effect for the trait of the clone in the plot and by the genotypic effect for competitive ability of clones in neighboring plots, as well as by the impact of that competition on the trait (Muir 2005). Thus, a statistical model can be used to estimate two genotypic effects: one for the trait and one for competitive ability. The incidence matrices for these genotypic effects differ: the incidence for the trait is to the clone in the plot while the incidence for competitive ability is to the clones in neighboring plots. If we assume that both the trait and competitive ability are primarily determined by additive gene action, then both genotypic effects have a covariance matrix proportional to the additive relationship matrix, though the additive genetic variance for the two traits will differ. In addition, there will be a genetic covariance between the two traits. Based on this hypothesis, a Model 1 can be built as follows:

$$Y_{n\times 1}=\mu +Z1_{n\times g}g_{g\times 1}+Z2_{n\times g}c_{g\times 1}+\epsilon \;1$$

$$\left[\begin{array}{c} g\\ c \end{array}\right]∼N\left(\begin{array}{cc} \left[\begin{array}{c} 0\\ 0 \end{array}\right], & \left[\begin{array}{cc} \sigma_{g}^{2} & \sigma_{gc}\\ \sigma_{gc} & \sigma_{c}^{2} \end{array}\right]⊗K_{g\times g} \end{array}\right)$$

$$\epsilon ∼N\left(0,I_{n\times n}\sigma_{e}^{2}\right)$$

where **Z2** is the incidence matrix for the genotypic effect for competitive ability whose construction will be discussed below; and ***c*** is the vector of competitive ability genotypic effects.

As for the measurement of the trait in the plot, there can be error in the estimate of the competitive ability coming from a neighboring plot. For example, a clone planted in an adjacent plot will exert less influence if it was established from weak propagules and therefore does not grow vigorously. Thus, as for the genetic effect, there can be error in the measurement of the trait and of the competitive ability, as well as a covariance between those errors. While modeling this scenario, the dimension of the incidence matrix is the as same as that of the residual error, but, unlike in residual error, the diagonal is zero because the incidence of this competition error is to the neighboring plots. A covariance between residual and competition errors can also be expected. In summary, the residual error (ϵ) from the Base model can be partitioned into a competition error and a residual error. Applying this idea to the Base model gives:

$$Y_{n\times 1}=\mu +Z1_{n\times g}g_{g\times 1}+II_{n\times n}p_{n\times 1}+I_{n\times n}r_{n\times 1}\;2$$

$$g∼N\left(0,K_{g\times g}\sigma_{g}^{2}\right)$$

$$\left[\begin{array}{c} p\\ r \end{array}\right]∼N\left(\begin{array}{cc} \left[\begin{array}{c} 0\\ 0 \end{array}\right], & \left[\begin{array}{cc} \sigma_{p}^{2} & \sigma_{pr}\\ \sigma_{pr} & \sigma_{r}^{2} \end{array}\right]⊗I_{n\times n} \end{array}\right)$$

where **II** is the incidence matrix for competition error whose construction is similar to that of **Z2**; ***p*** is the vector of competition errors; ***r*** is the vector of residual errors; and **K** is the genomic relationship matrix.

A full model accounting for the direct and competitive genotypic effect, and the competition error results from combining models 1 and 2:

$$Y_{n\times 1}=\mu +Z1_{n\times g}g_{g\times 1}+Z2_{n\times g}c_{g\times 1}+II_{n\times n}p_{n\times 1}+I_{n\times n}r_{n\times 1}\;3$$

$$\left[\begin{array}{c} g\\ c \end{array}\right]∼N\left(\begin{array}{cc} \left[\begin{array}{c} 0\\ 0 \end{array}\right], & \left[\begin{array}{cc} \sigma_{g}^{2} & \sigma_{gc}\\ \sigma_{gc} & \sigma_{c}^{2} \end{array}\right]⊗K_{g\times g} \end{array}\right)$$

$$\left[\begin{array}{c} p\\ r \end{array}\right]∼N\left(\begin{array}{cc} \left[\begin{array}{c} 0\\ 0 \end{array}\right], & \left[\begin{array}{cc} \sigma_{p}^{2} & \sigma_{pr}\\ \sigma_{pr} & \sigma_{r}^{2} \end{array}\right]⊗I_{n\times n} \end{array}\right)$$

**Figure 1:**
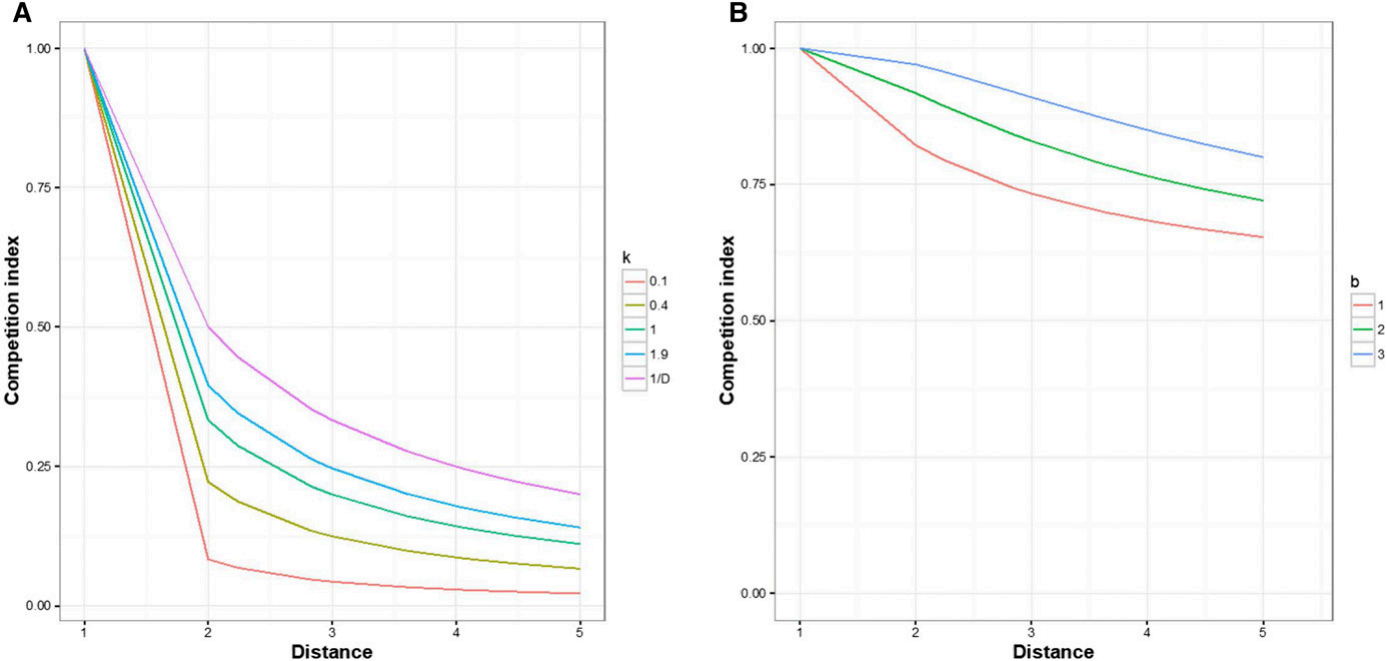
Competition index depicted for FD (A) and SD (B) functions with respect to distance in meters. For FD, the competition reduces to <25% when the distance between plots is >5 m, which usually is the distance between adjacent columns in the real data study. For SD, the intensity of competition is assumed to decay slowly and is conditionally terminated at a certain distance. In the depiction here, it is terminated at 5 m and still have >60% intensity. Parameters *k* and *b*, which were used to generate FD and SD functions, respectively, are represented with different colors.

The construction of **Z2** and **II** follows from these considerations. The ability of a cassava clone to influence the growth of neighbors need not necessarily be limited to the nearest neighbor plot as roots can forage for water and nutrients, and shade can be cast further than that (Izumi *et al.* 1999). We are not aware, however, of studies that evaluate the rate of decay of competitive effects with distance, and we wanted to allow for fast or slow decay. To account for all these possibilities, we used three different functions relating distance to the extent of competition: nearest neighbor (NN), fast decay (FD), and slow decay (SD). The NN was the simplest, and we tested two NN functions: (i) only the neighbors along the longest edge of the rectangular plot were considered, and they were given an incidence of 1; and (ii) all the neighbors were considered with neighbors on longer edge given an incidence of 1, neighbors on the shorter edge an incidence of 0.5, and diagonal neighbors an incidence of 0.2. The FD and SD incidences were smooth functions of the distance between plot centers:

$$FD=\frac{k\left(\frac{1}{D}\right)}{\left(k-\frac{1}{D}\right)+1}$$

$$SD=\frac{1}{c+b^{\frac{1}{D}}}$$

where D is the distance between plots; *k* is the FD parameter whose values ranged from 0.1 to 2 to accommodate different competition intensities as the distance between neighbors increases (Figure 1A). When *k* = 0.4, the incidence matrix is similar to the square of inverse of distance (Muir 2005). The SD function is a modified logistic function where parameters *c* and *b*, respectively, takes a value from a pair set containing pairs of values as {(0.9,0.1), (0.99,0.01),…} (Figure 1B). The FD function assumes that the competition fades considerably beyond the nearest neighbors, but can continue feebly as the distance increases. The SD function assumes that competition fades slowly and its intensity can remain high and stable. The SD function is also modified such that competition stops beyond a specified distance. Use of distance-based competition functions and exploratory model fitting both contribute to account for missing/dead plants. Specifically, the deviation in the competition effect from a plot relative to what would be expected had there been no missing/dead plants is captured in the competition error term, irrespective of the genotype.

### Simulation studies

Because the competition model is complicated, we discuss the approach we used to simulate competitive effects before we discuss analysis of real data. This discussion will serve to highlight intricacies of the statistical model that needs to be fit. We conducted a simulation study to test the ability of the GS-competition Model 3 to (a) reduce error, (b) correctly partition phenotypic variance, and (c) improve accuracy of the estimation of genetic effects of measured traits. A dataset containing 829 genotypes, including 11 check genotypes and their field coordinate information, was used to start the simulations. A genomic relationship matrix (**K**) was also provided for the dataset in use.

Trait genotypic effects were simulated with zero mean and unit variance. Values for competition genotypic variance ($\sigma_{c}^{2}$), correlation between trait and competitive genotypic effect (Gcor), trait genotypic ratio (*gr*, see below), correlation between competition error and residual error (Ecor), and fraction of competition error (fraE) were also provided as simulation parameters. A genetic variance-covariance matrix (**G**) was produced using Gcor, and the two genotypic variance values:

$$\sigma_{gc}=Gcor\left(\sqrt{1*\sigma_{c}^{2}}\right)$$

$$G=\left[\begin{array}{cc} \sigma_{g}^{2} & \sigma_{gc}\\ \sigma_{gc} & \sigma_{c}^{2} \end{array}\right].$$

Calculation of genotypic effects was broken down in the following fashion. First, the Cholesky decomposition of **G** (chol.G) and **K** (chol.K) matrices was performed. Second, two separate random effect values (geno) following normal distribution of mean 0 and SD of 1 were generated for each genotype. Third, genotypic effects (geno.G) were calculated using chol.G matrix as following:

$$geno.G=chol.G^{T}*geno$$

Fourth, the geno.G values for direct and competitive genotypic effect were multiplied by the chol.K matrix in order to calculate the genotypic effects in the presence of **K** matrix, as follows:

$$geno.Effects=chol.K^{T}*geno.G^{T}$$

The genotypic effects matrix can be separated into direct and competitive genotypic effects. The genotypic effects were calculated for low and high direct genotypic ratio scenarios. The direct genotypic ratio (*gr*) can be formulated as

$$gr=\frac{\sigma_{g}^{2}}{\sigma_{g}^{2}+\sigma_{c}^{2}+\sigma_{e}^{2}}.$$

With trait genotypic effect fixed at unit variance, the error variance can be calculated as

$$\sigma_{e}^{2}=\left(\frac{\left(1-gr\right)}{gr}\right)-\sigma_{c}^{2}.$$

The fraE value was used to partition the total error variance into that for competition error and residual error:

$$\sigma_{p}^{2}=fraE*\sigma_{e}^{2}$$

$$\sigma_{r}^{2}=\left(1-fraE\right)*\sigma_{e}^{2}.$$

Later, the error effects were calculated in a same way as the geno.G effects, producing separate vector of effects for competition and residual errors.

Afterward, the **Z2** matrix was created using the distance-based functions. Two different competition functions were used to simulate the **Z2** matrix–NN for neighbors along the longer edge of a rectangular plot and SD. NN was chosen as the simplest form of competition and SD was chosen as it turned out to represent most instances in the real data study. The distance between plots was calculated on the assumption that plot dimension was 2 × 1. The **II** matrix was created using NN function for neighbors along the longer edge of the rectangular plot. In order to calculate the phenotypic expression, the **Z1**, **Z2**, and **II** matrices were multiplied by the direct effect, competitive effect, and competition error effect, respectively. The resulting values were added to the residual error values to calculate the phenotype.

The variance of the competitive ability effect was considered to vary from 0.4 to 0.1 when direct genotypic ratio values were high (0.7), and to vary from 1 to 0.1 when the ratio values were low (0.3). Two scenarios of correlation between direct and competitive ability genotypic effect were considered: (i) no correlation and (ii) a correlation of 0.4. In scenarios where competitive genotypic effect is very high, the variance due to it contributes mostly to the total genotypic variance. To illustrate this, consider the scenario of unit variance for both direct and competitive genotypic ability. When it comes to phenotypic expression, the competitive influence on the neighboring plots is calculated as indicated by the **Z2** matrix. Because a plot has multiple neighbors, multiple competitive effects may affect it, leading to a high impact of the competitive ability variance compared to the trait genotypic variance, even in the relatively simple NN scenario.

Two scenarios of correlation between competition and residual error effects were accounted for: (i) no relation and (ii) a high positive correlation of 0.8. A combination of values ranging from 0.3 to 0.9 corresponding to the fraction of competition to total error variance was used simulate the competition and residual error effects. The combination was used to determine the variance of competition and residual error effects.

Three scenarios for clone replication were considered in the simulation. First, the scenario with minimum replication contained only the checks replicated at least twice with all the test clones represented once. Second, 50% of the test clones were replicated twice in addition to the presence of replicated check clones. A third scenario was considered where all clones were replicated at least twice.

The GS-competition Model 3 with **Z2** matrix modified using NN, FD, and SD competition functions was tested along with the Base model on simulated data. The simulation and analysis was repeated 20 times. The best model was selected as the one with the lowest root mean squared error (RMSE), *i.e.*, the mean squared deviation between the estimated and the simulated genotypic effect. Accuracy (the correlation between true and estimated genotypic effects) was also used as a criterion for selecting these models.

### Real data studies

Model 3 and its two variants (Model 1 and 2) were used to evaluate potential competition effects in real data. A 10-fold cross validation (CV) repeated five times was used. The best model was chosen as the one with the lowest prediction root mean squared error (pRMSE) between observed (Y) and predicted (Ŷ) response values for the test dataset. Models using various incidence matrices to account for competition were compared to the Base model having no competition component, and a model was selected based on its predictability and significance. Details on the CV method are given in the companion paper (Elias *et al.* 2018). Relative reduction in pRMSE was calculated as the ratio of difference in model pRMSE to Base pRMSE. Values for prediction correlation (pCOR or accuracy) were also recorded for the best model as the correlation between observed and calculated response values for the test dataset. Relative increase in pCOR was calculated as the ratio of difference in pCOR to 1–Base pCOR. The relative difference values were multiplied by 100 to convert them to percentages.

**Figure 2:**
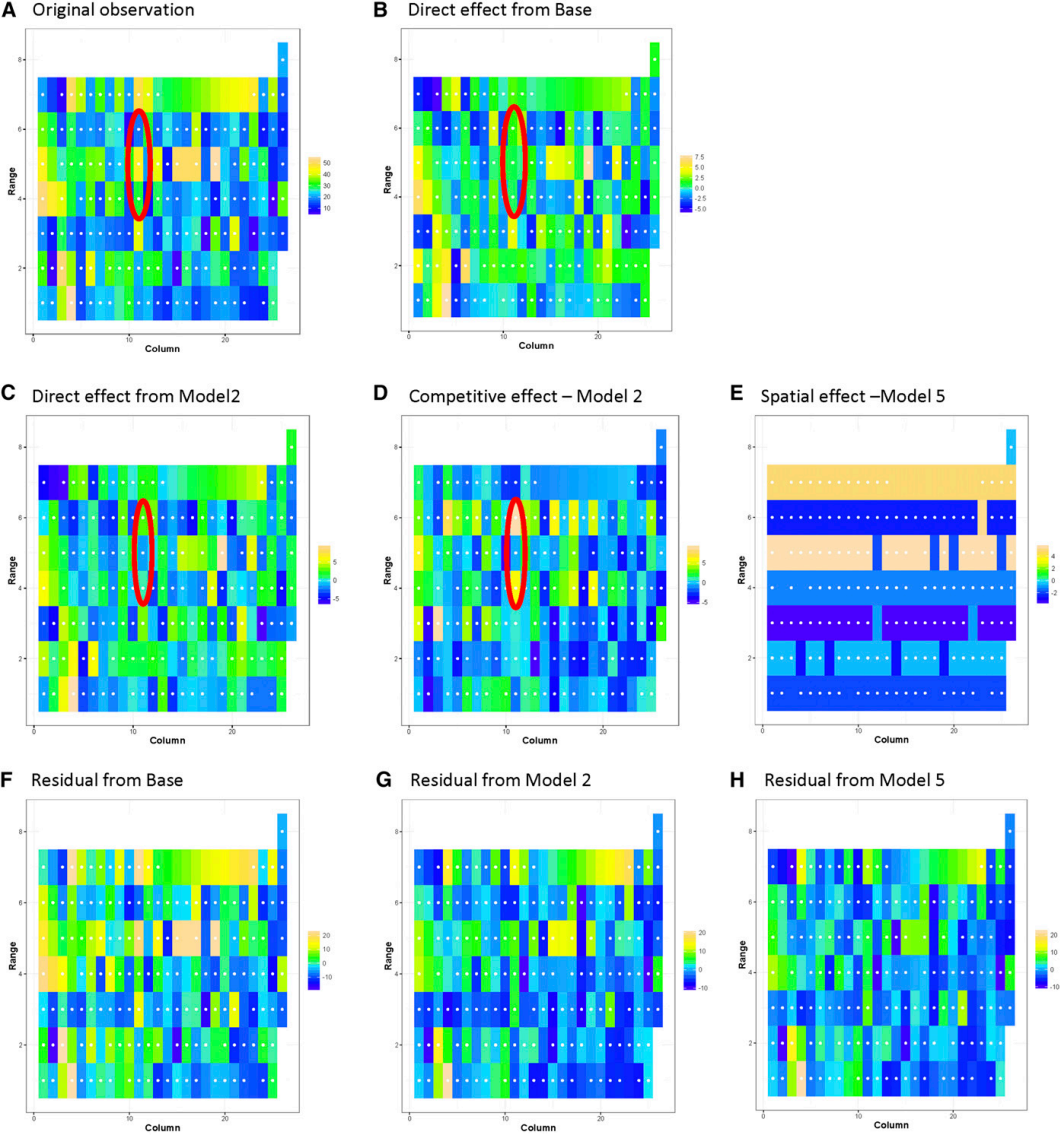
Comparison of direct, competitive, and spatial effect on shoot weight of Ibadan_2014_PYT as illustrated by (A) original observation, (B) direct effect from Base, (C) direct effect from Model 2, (D) competitive effect from Model 2, (E) spatial effect from Model 5, (F) residual from base, (G) residual from Model 2, and (H) residual from Model 5. The genotype in the target plot present in Range 5 and column 11 (inside the red oval) having weak direct genotypic effect (C) has high shoot weight value (A) due to relatively low NN competitive effect (D). The target plot, however, exhibited a relatively strong competitive influence on its NN neighbors resulting in their lower shoot weight. Residual values are modified in the presence of significant competitive and spatial effects (F–H).

In our companion paper (Elias *et al.* 2018) we used a GS-spatial model (hereafter called Model 4) to evaluate potential spatial correlation in these field trials:

$$Y_{n\times 1}=\mu +Z1_{n\times g}g_{g\times 1}+I_{n\times n}s_{n\times 1}+I_{n\times n}r_{n\times 1}\;4$$

$$s∼N\left(0,S\sigma_{s}^{2}\right),$$

where **s** is the vector of spatial effect and **S** is the selected spatial correlation structure for the trait. In this paper, the selected GS-competition model from among Model 1, 2, or 3 for a trait was updated as Model 5 if a significant spatial component from Model 4 was found. An illustration of the impact of direct genotypic effect, competitive effect on target plot and residuals can be found in Figure 2.

The data were fitted using the “regress” package in R v. 3.2.5 using restricted maximum likelihood (REML). A Chi-square test with a significance threshold of 2.706 at *α* value of 0.1 (Stram and Lee 1994) was used to test the significance of the additional variance component in the selected model compared to the base. The threshold value was changed with degrees of freedom. For example, on comparing Base with Model 5 having two extra components, the threshold was taken as 4.605 at *α* value of 0.1. Trait heritability was calculated for the selected model, and compared with Base based on the approach introduced by Cullis *et.al*. (2006) as follows:

$$h^{2}=1-\frac{{\hat{V}}_{BLUP\;difference}}{2{\hat{\sigma }}_{g}^{2}}$$

where ${\hat{V}}_{BLUP\;difference}$ is the mean variance of the difference across all pairs of genotypic BLUPs; ${\hat{\sigma }}_{g}^{2}$ is the estimated variance of genotypes.

Total phenotypic variance was calculated as the sum of variance values from different components of the model after analysis. The datasets and SNP files used for performing this study can be found in the following link: ftp://ftp.cassavabase.org/manuscripts/Elias_et_al_2017_datasets/.

### Automation of real data analysis, simulation of data, and its analysis

Functions were written to automate the real and simulated data analysis. For the real data analysis, the minimum requirements for the function are a .csv file having field coordinates (Ranges and Columns), trait(s), and clone names, and a genotypic relationship matrix based on marker or pedigree data. Providing a plot dimension (width × length to calculate distance between ranges and columns) can help to identify the best model. The function can take care of the initial processing of the dataset including removal of missing values for a particular trait, matching the genotypes with those in the relationship matrix, and removal of outlier points whose residuals are >2.5 times the error SD (after testing using the Base model). The output of the algorithm will be saved in the working directory of R. The output will contain the predictions for direct and competition genotypic effect, pRMSE, and predicted correlation (pCOR) values, number of failed models as trapped by the “try” function in R during the evaluation of a model, and summaries of the Base and the selected model including the parameter values (if the selected model is different from the Base). For the simulations, a dataset with genotypes and field coordinates must be given along with different parameter values, such as correlation of direct to competition effect, correlation of competition to residual error, genotypic ratio, competition variance, fraction of competition to total error, and plot dimension. The output contains a .csv file of RMSE and correlation between true and estimated genotypic direct and competition effects, error effects, fraction of competition error, genotypic ratio, and number of models failed. The functions are available in the following web link: ftp://ftp.cassavabase.org/manuscripts/Elias_et_al_2017_competition.zip.

### Data availability

Tables S1–S4 in File S1 contains ANOVA tables from analyzing the simulated data. The legend of Table S1 in File S1 compiles the parameter values used for simulation studies. Table S5 in File S1 provides information on the best model selected after CV in all the trial-trait scenarios. Table S6 has information on scenarios where Model 5 fit best. Figure S1 in File S1 shows the original observation, direct genotypic BLUP, and competitive effect from Model 2 or 3 for all the trial-traits mentioned in Table 1 except for that in Figure 4.

## Results

### Simulation studies

We showed from our real data studies that estimates of the trait genotypic effect are affected even when the competition variance was small (<10% of the total variance in most of the scenarios). Therefore, we conducted simulation studies to see the impact of instances where competitive variance would be larger (10–100 of the trait genotypic effect.

**Figure 3:**
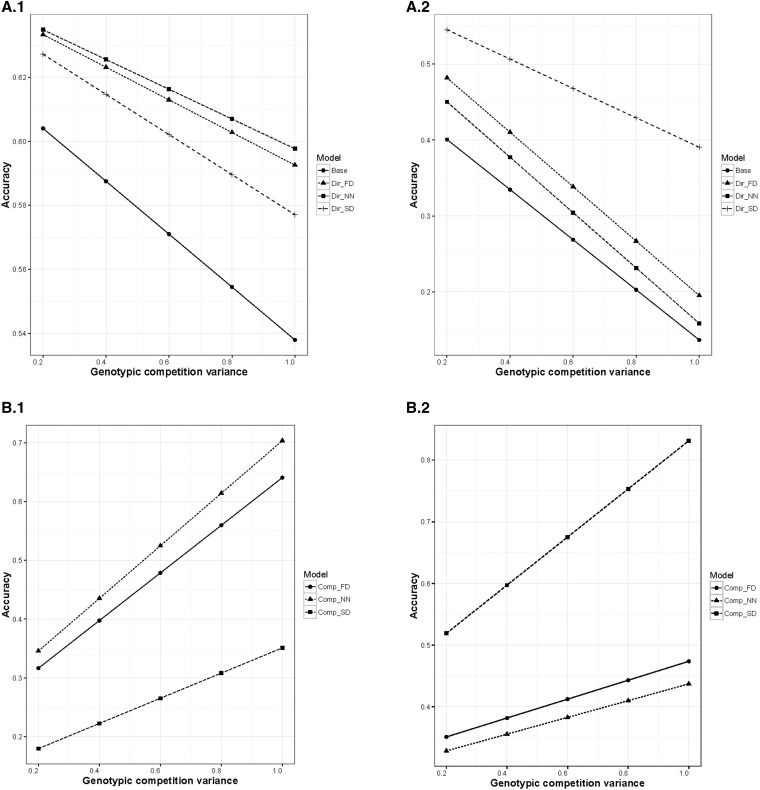

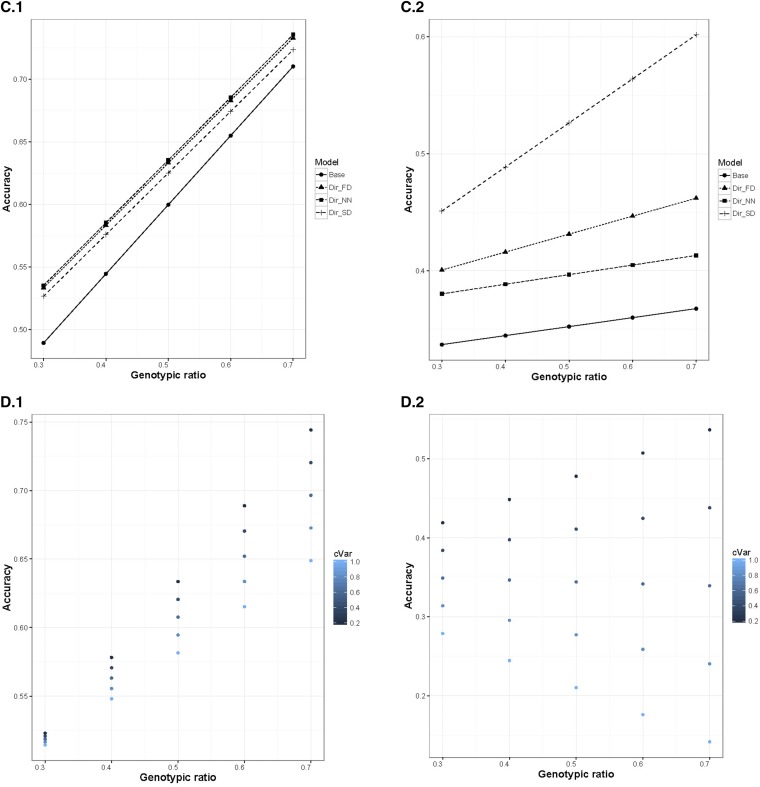
Correlations (accuracy) between true and estimated genotypic values from simulations. ANOVA tables are given in supplemental section. The Base model only estimates the direct genotypic effect, while _FD, _NN, and _SD models also estimate genotypic and error competition effects analyzed using FD, NN, and SD competition decay functions. The prefixes Dir or Comp indicate that the correlations are for the trait or the competition effect, respectively. Rows A and B show the impact of the genotypic competition variance on accuracy of direct genotypic effect (A) and competition genotypic effect (B) averaged over trait genotypic ratio. Rows C and D show the impact of trait genotypic ratio on trait genotypic accuracy, as affected by analysis model (C) or genotypic competition variance (D). Plots in the first (left hand) and second (right hand) columns show analyses of data simulated with the NN and SD competition decay functions, respectively.

The model fitting the function with which the data were simulated outperformed other models in all the scenarios (Figure 3). The accuracy in estimating the trait genotypic effect was increased by a median value of 9% compared to Base while evaluating a NN simulated dataset (Figure 3A.1). This accuracy was increased by 26% if the underlying SD competitive effect was correctly identified (Figure 3A.2). However, with an increase in competition variance, the accuracy for predicting the trait genotypic effect for all the models diminished with relatively less reduction for the best model (Figure 3A). The contribution of competitive variance component to the total genotypic variance can be higher than that of the trait genotypic effect with increase in competitive variance. This higher contribution is because of the cumulative competitive influence from neighbors, as accounted for by the **Z2** matrix. In estimating the trait genotypic effect, these neighbor effects are essentially a source of noise that becomes stronger as the competitive variance increases. Conversely, an increase in competitive variance in the simulated data resulted in a corresponding increase in accuracy for predicting the competitive effect (Figure 3B).

When data were simulated with NN, modeling with the SD function exhibited the lowest accuracy (excluding Base) and vice versa (Figure 3, A and B). The FD function performed similarly to the NN function, while the SD function remained distinct from the rest of the models (Figure 3, A and B). The function NN assumes that competitive influence can only occur between adjacent neighbors, and, thus, that it decays rapidly, like the FD function (Figure 1A). This property made the NN and FD functions behave similarly. The robustness of FD can be justified as both FD and SD functions accommodated competition beyond the nearest neighbor. The competitive influence between adjacent plots was same for SD and FD functions. However, in SD, the influence beyond the adjacent neighbors decreased slowly, and never diminished to <60%. This property made the SD function distinct (Figure 3 column 2), and poorly approximated by other functions. From our real data studies, we found that non-NN functions explained the competition variance in most of instances, and that the SD function was slightly more frequent.

The accuracy of direct and competition genotypic effect for Model 3 can be increased with increase in number of replications for genotypes (Tables S1–S4 in File S1). Increase in genotypic ratio was advantageous to Model 3, and clear distinction was observed while analyzing data where true competition followed a SD pattern (Figure 3C). Influence of increase in competition variance while simulating the data with increasing genotypic ratio can be visualized in Figure 3D. A higher value for accuracy was observed in all the instances where competition variance is low. Accuracy increased with increase in genotypic ratio where the increase was more profound for low variance in data simulated with NN. In the case of data simulated with SD, accuracy decreased with increase in genotypic ratio for high competition variance, and increased for low competition variance.

### Real data studies

We tested for genotypic competition in nine trials and four traits. In 12 of the 36 combinations a significant competition effect was observed (Table 1 and Table 2). A significant genotypic competition effect was observed for one or more of the traits tested in all the trials except for C2 trials in Ikenne and Mokwa (Table 1 and Table 2). The competition variance in two out of 12 cases was better explained as competition error. On trials with large numbers of genotypes (C1 trials from all locations with >600 genotypes), more than one trait showed a significant competition effect.

**Table 2:** Summary of results from prediction analysis using real datasets comparing base to GS-competition model for all the traits under study where significant competition effect at *α* = 0.1 was observed, out of 36 trial/trait combinations

Trait	Type	# of Trials	Reduction in pRMSE (%)	Increase in pCOR (%)
DM	NNc	1	1	2.8
	NNp	1	NA	NA
	FD	1	0.1	0.3
	SD	1	NA	NA
HI	NNc	1	0.8	3.3
	NNp	1	NA	NA
	FD	2	0.6	2.5
	SD	1	1.8	7.6
FYLD	NNc	1	NA	NA
	NNp	1	1.5	7.4
	FD	1	NA	NA
	SD	2	0.7	5.5
SHTWT	NNc	1	NA	NA
	NNp	1	4.5	25.2
	FD	1	NA	NA
	SD	2	0.9	3.8

NNc, NN-genotypic competition; NNp NN-competition error.

We observed a reduction in pRMSE as high as 4.5%, and an increase in pCOR as high as 25% when significant competition was explained by Model 1 or 2 (Table 2). The results also showed that the interplot competitive effect was not limited to the NN. The NN function best explained competition in two cases while a non-NN function did better in the remaining eight cases (Table 1 and Table S5 in File S1). Usage of distance based non-NN function also helped to identify the distance at which the competition stopped or diminished markedly (Figure 1, Table 1, and Table S5 in File S1).

Results from Model 2 (Table 1 and Table S5 in File S1) also showed that accounting for interplot competition modified the trait genotypic variance and reduced the error variance. An exceptional scenario was found in Ikenne_2014_C1 for DM, which can be an artifact of the competition function. In this case, the residual variance was found to be negligible, and all the variance in the phenotype was explained by direct or competition genotypic effect, even though the competition explained only 2.6% of total variance. The variance explained by interplot competition component was <10% in all of the scenarios.

Heritability of the traits ranged from 0.27 to 0.84 (Table 1). Slight or no modifications in heritability was observed when competitive ability was accounted for in the model. An exception was the 13% decrease in heritability observed in the PYT trial for SHTWT.

The trait genotypic effect was moderate to strongly positively correlated to observed phenotypic value, while the competition effect was weakly correlated to it. The competition effect was also weakly correlated to the trait and residual effects. The exception to this behavior was HI and DM from 2014_C1 trials conducted in Ibadan and Ikenne, exhibiting a moderately negative correlation between competition and residual effects (Table S5 in File S1). Weak correlation between genotypic trait and competition variance indicated that not all the highly productive genotypes were aggressive, and not all the high aggressive genotypes were highly productive.

Strong positive correlation (0.7) between above- and below-ground competition effect in Ibadan_2013_C1 indicated that the competitiveness was similar for the whole plant in these genotypes. In Ikenne_2013_C1 moderate positive correlation (0.45) between above- and below-ground impact of competition for the genotypes was observed. The HI showed most instances of a significant competition effect. Significant competition in HI indicated that cassava could change its biomass allocation strategy when influenced by a neighboring competitive genotype. The competition effect, which explained only a very small percentage of variance in phenotype, was, however, weakly correlated with the trait genotypic effect in all the traits. However, all the trials with significant competitive effects exhibited it for below ground biomass where the harvest product of interest was located.

**Figure 4:**
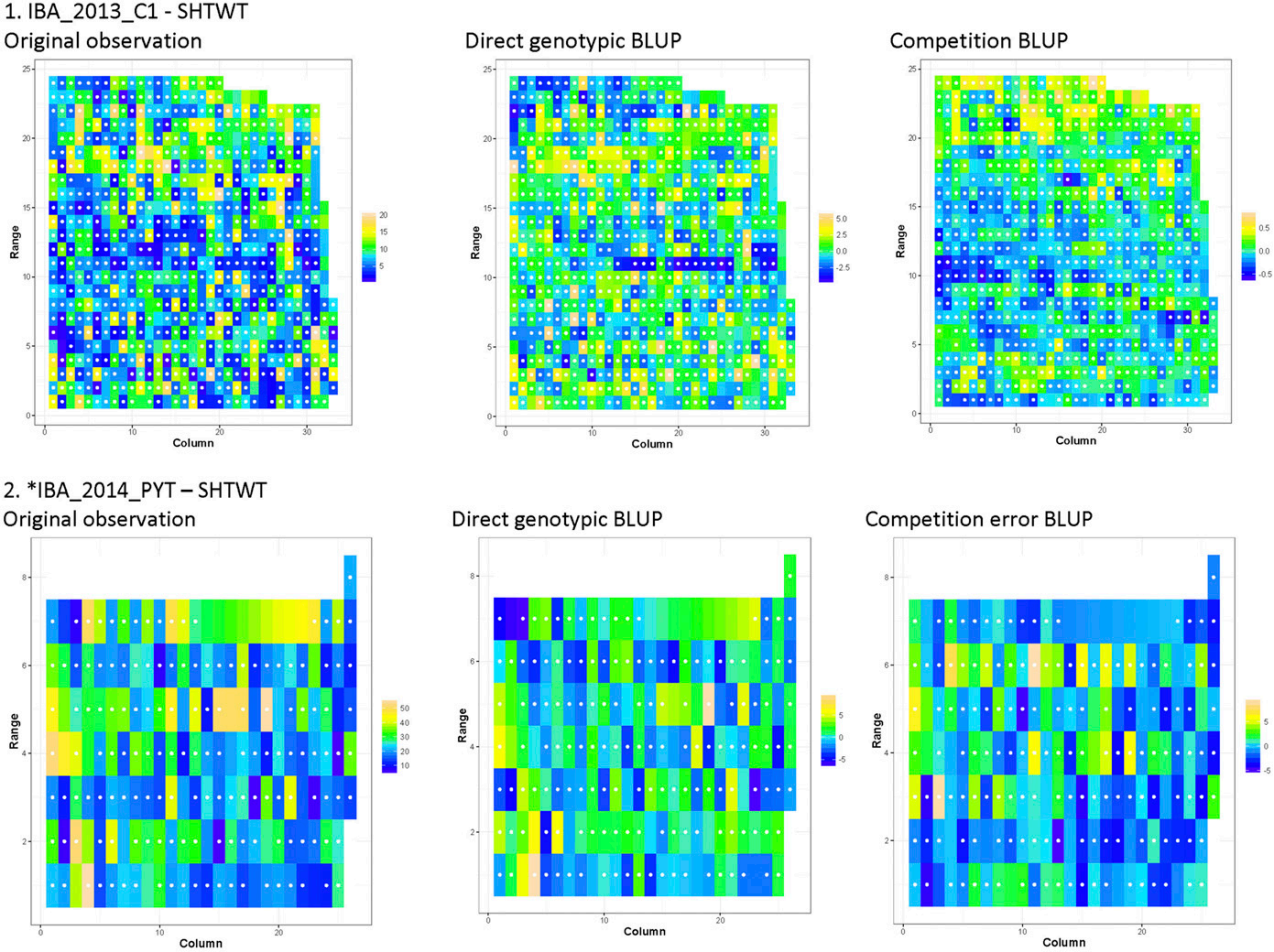
Original observation (column 1), direct genotypic BLUP (column 2), and competition BLUP OR competition error BLUP* (column 3) from model for SHTWT illustrated from (1) IBA_2013_C1 and (2) *IBA_2014_PYT. Visualization of values from all other trial-traits that exhibited significant competitive effect can be found in Figure S1 in File S1. Missing values were linearly interpolated.

From the visual assessment (Figure 4 and Figure S1 in File S1), no pattern of border effect with similar colored plots around the edge of the field was evident. This observation was consistent for observed phenotypic values, and for both of the genotypic effects. The genotypic effects were distributed randomly and among the family of genotypes.

In Mokwa_2014_C1 FYLD (Figure S1C.3 in File S1) and Ibadan_2014_PYT SHTWT (Figure 4.2), the variance due to competition was explained by the competition error. In Mokwa plots with low competitive ability placed together across the Ranges and Columns could be contributing to the low FYLD values. Some evidence of spatial correlation can also be found for this particular trait (Elias *et al.* 2018). In Ibadan, low trait genotypic effect paired with low competitive ability might have contributed to low SHTWT. Strong evidence for the coexistence of interplot competition and spatial correlation was observed for this particular trait (Table S6).

We analyzed these data for the same traits to evaluate the presence of spatial correlation (Elias *et al.* 2018), and we used that information to build Model 5. There were four trials that exhibited significant spatial and competition effect with a significant improvement of the spatial model over the Base or competition model (Table S6). All the trial/traits except in Ibadan_2014_C1 HI, interclonal competition and spatial correlation along with trait genotypic variance explained significant variance in the phenotype. In Ibadan_2014_C1 HI, accounting for competition variance along with trait genotypic variance was found to be better in explaining the variation present in the phenotype.

## Discussion

This study applies a GS model that accounts for variance among genotypes for competitive ability for the first time. These analyses use non-iid relationship matrices—an additive genomic relationship matrix based on SNP markers for trait and competition genotypic effects. Using this approach, we found that the predictive ability of the GS model increased significantly even in the absence of replication for test genotypes. Our simulation studies indicated that the predictive ability of GS models for competitive as well as trait genotypic ability can increase with an increase in number of replications.

Another innovative element in this study is considering the competition of a genotype beyond its nearest neighbor. Competitive ability is the genetically determined ability of an individual to influence its neighbors. This may be unrelated to productivity or to genotypic effect for other traits. Cassava’s rapidly growing and highly proliferating adventitious and lateral roots can penetrate deep and extensively in search of nutrients and water (Cock 1985; Izumi *et al.* 1999; Pardales and Yamauchi 2003). This growth can influence the neighbors beyond the nearest ones, as in the case of a highly competitive clone whose roots can encroach into the root zone of a noncompeting neighbor. The extensive nature of cassava roots could also be explain why no distinct pattern of border effect was evident from our field studies. Competition of a clone beyond the nearest neighbor could also result from a diminishing domino effect, where neighbors beyond the nearest ones are indirectly influenced: a competitive clone might cause its neighbors to extract resources away from it, thereby leading them to encroach more on their neighbors. The proposed GS competition models can identify significant competition, and estimate its variance even when randomization is restricted. Restricted randomization was applied in the field study to simplify evaluation of clones within families by keeping clones from the same family together. This type of design is also expected to decrease the bias due to competition (David *et al.* 1996). With the information from our analysis this design can be modified by not placing genotypes with marked differences in competitive ability near each other.

A third innovative application of our model is the consideration of plot orientation and dimension. In most of our field studies, plants were arranged in plots with single rows where the longer edge of the plot was shared among ranges. The effect of competition is expected to be higher on the neighbors along the longer edge (along the Range) compared to that on the shorter edge (along the Column). This difference was accounted for in the distance-dependent competition functions. However, we have no information on the intraplot competition in multiple row plots of the same clone.

All plants in a single row plot might be able to use the water and nutrients provided in the interplot space fairly equally. Moreover, cassava being a clonally propagated plant, the portion of stem used as planting material could also determine the amount of interplot competition. A plot planted with vigorous stem cuttings might capture more resources than the same clone planted with less vigorous cuttings. To account for this source of error, we included a competition error term—a fourth innovation in the GS models. Understanding the competition error could improve the choice of planting material to mitigate errors in performance due to propagule vigor.

The genotypes selected based on trait and competitive genetic effects can be used for the next cycle of breeding or for commercialization. When spatial correlation is present, it should be adjusted using the proposed model before selecting the elite genotypes. The trait genotypic effect should be the first component to consider while selecting, as performance was moderate to strongly positively correlated to it. Competition variance for genotypes were <10% of their trait genotypic variance in most the scenarios in real data studies. During such instances, genotypes with high trait genotypic effect (after adjusting for the competition effect) can be selected as elite. Therefore, a wise selection for yield increase would be that with high direct and moderate competitive ability. If highly competing plants are selected, they may decrease whole plot performance (Donald 1968; Kempton 1982). Selecting less competitive genotypes in order to increase the yield per unit area is effective if the trait and competitive effects of genotypes are negatively related (Cannell 1979). In our real data studies, we observed that the competition effect is weakly or negatively correlated to the trait genotypic effect. Muir (2005) suggested a linear index for selecting individuals when trait and competition effects are present as a weighted sum of these two effects. The index showed that, even in scenarios where the competition effect was small, its contribution could be higher than the trait effect in large groups of individuals.

### Conclusion

Through real data studies, we showed that a small but significant increase in predictive ability was attained when interclonal competition was taken into account in a GS model. This predictive ability was markedly increased when accounting for the competition error if present. Results from simulation studies indicated that noticeable increases of accuracy in predicting the breeding value could be attained. These increases occurred especially when competitive influence reached beyond the adjacent neighbors and was accounted for in the GS model. Knowing the competitive ability of a genotype can help in selecting genotypes for breeding advancement. This knowledge can also be useful in modifying the spatial arrangement when genotypes with marked differences in competitive ability need to evaluate together in clonal evaluation trials. This knowledge can also facilitate adjusting the timing and quantity of irrigation and nutrient application, and application of management practices such as pruning and trimming.

## Supplementary Material

Supplemental material is available online at www.g3journal.org/lookup/suppl/doi:10.1534/g3.117.300354/-/DC1.
