# Supplementary material for: Improving Genomic Prediction in Cassava Field Experiments by Accounting for Interplot Competition
Source: G3 (Bethesda). 2018 Jan 22;8(3):933–44. doi: 10.1534/g3.117.300354 (PMC5844313; doi:10.1534/g3.117.300354)

Supplementary Tables

Table S1. ANOVA table of the final model after step-wise regression selecting the best fitting model based on Akaike Information Criterion (AIC) for data simulated with NN competition function. Correlation (COR) between true and predicted values of direct genotypic effect is the response variable. Explanatory variables are Model (various models such as Base (with direct genotypic effect only), Dir_NN (with direct and NN competition genotypic effect), Dir_SD (with direct and SD competition genotypic effects), Dir_FD (with direct and FD competition genotypic effect)), fraE (fraction of error variance: 0.3, 0.6, and 0.9 are the three values used), Gcor (correlation between direct and competition effects: 0 and 0.4 are the two values used), Ecor (correlation between competition and residual error effects: 0 and 0.8 are the two values used), h2 (direct genotypic ratio 0.3 and 0.7 are the two values used), cVar (competition genotypic variance: values ranging from 0.1 to 1), rep indicates the percentage of test genotypes replicated. Explanatory variables are same for all the ANOVA tables from simulation.

Analysis of Variance Table

Response: COR

	Df	Sum Sq	Mean Sq	F value	Pr(>F)	
Model	3	4.094	1.365	334.4446	< 2.2e-16	***
fraE	1	0.022	0.022	5.3439	0.0208052	*
Gcor	1	0.059	0.059	14.4961	0.0001409	***
Ecor	1	0.000	0.000	0.0265	0.8705954	
h2	1	222.346	222.346	54489.0978	< 2.2e-16	***
cVar	1	0.901	0.901	220.7939	< 2.2e-16	***

```
rep            1  77.457  77.457 18982.0652 < 2.2e-16 ***
Model:fraE     3   1.026   0.342    83.8324 < 2.2e-16 ***
Model:Gcor     3   0.111   0.037     9.0967 5.163e-06 ***
Model:Ecor     3   0.030   0.010     2.4147 0.0645415 .
Model:h2       3   0.725   0.242    59.2277 < 2.2e-16 ***
Model:cVar     3   0.290   0.097    23.6660 2.779e-15 ***
Model:rep      3   0.048   0.016     3.9256 0.0082004 **
fraE:rep       1   0.138   0.138    33.8754 5.968e-09 ***
h2:rep         1   2.166   2.166   530.8690 < 2.2e-16 ***
cVar:rep       1   0.243   0.243    59.5233 1.267e-14 ***
fraE:h2        1   0.170   0.170    41.7213 1.078e-10 ***
fraE:cVar      1   0.055   0.055    13.4513 0.0002455 ***
Gcor:cVar      1   0.022   0.022     5.3539 0.0206863 *
Ecor:h2        1   0.143   0.143    35.1451 3.112e-09 ***
h2:cVar        1   1.474   1.474   361.1118 < 2.2e-16 ***
Residuals  19404  79.179   0.004
---
Signif. codes:  0 ***,0.001 **,0.01 *,0.05 .
```

Table S2. ANOVA table of the final model after step-wise regression for data simulated with SD competition function. Correlation between true and predicted values of direct genotypic effect is the response variable. Explanatory variables are same as those described in Table S1.

Analysis of Variance Table

Response: COR

	Df	Sum Sq	Mean Sq	F value	Pr(>F)	
Model	3	65.342	21.781	3354.6369	< 2.2e-16	***
fraE	1	0.057	0.057	8.7162	0.0031581	**
Gcor	1	1.149	1.149	176.9584	< 2.2e-16	***
Ecor	1	0.791	0.791	121.8203	< 2.2e-16	***
h2	1	84.003	84.003	12937.9872	< 2.2e-16	***
cVar	1	53.728	53.728	8275.1304	< 2.2e-16	***
rep	1	25.232	25.232	3886.2238	< 2.2e-16	***
Model:fraE	3	0.117	0.039	6.0307	0.0004228	***
Model:Gcor	3	4.621	1.540	237.2658	< 2.2e-16	***
Model:Ecor	3	0.010	0.003	0.5306	0.6612864	
Model:h2	3	5.898	1.966	302.8051	< 2.2e-16	***
Model:cVar	3	6.352	2.117	326.1190	< 2.2e-16	***
Model:rep	3	1.134	0.378	58.2329	< 2.2e-16	***
fraE:rep	1	0.022	0.022	3.3651	0.0666079	.
Gcor:rep	1	0.445	0.445	68.6034	< 2.2e-16	***
Ecor:rep	1	0.010	0.010	1.5251	0.2168590	
h2:rep	1	0.610	0.610	93.9223	< 2.2e-16	***
cVar:rep	1	1.342	1.342	206.7244	< 2.2e-16	***
fraE:Ecor	1	0.030	0.030	4.6300	0.0314301	*
Gcor:Ecor	1	0.000	0.000	0.0141	0.9054234	
Gcor:cVar	1	0.265	0.265	40.8739	1.664e-10	***
Ecor:h2	1	0.072	0.072	11.0572	0.0008852	***
Ecor:cVar	1	0.077	0.077	11.8465	0.0005790	***

```
h2:cVar        1  11.190  11.190  1723.4452 < 2.2e-16 ***
Residuals  17721 115.057   0.006
---
Signif. codes:  0 ***,0.001 **,0.01 *,0.05 .
```

Table S3. ANOVA table of the final model after step-wise regression for data simulated with NN competition function. Correlation between true and predicted values of competition genotypic effect is the response variable. Explanatory variables are same as those described in Table S1. The variable Model has only three factors – Comp_NN (with NN competition genotypic effect), Comp_SD (with SD competition genotypic effect), and Comp_FD (with FD competition genotypic effect).

```
Analysis of Variance Table

Response: COR
              Df  Sum Sq Mean Sq    F value    Pr(>F)
Model          2 113.643  56.822  8098.3487 < 2.2e-16 ***
fraE           1   0.774   0.774   110.3361 < 2.2e-16 ***
Gcor           1   0.395   0.395    56.3460 6.430e-14 ***
Ecor           1   0.161   0.161    22.8881 1.734e-06 ***
h2             1   7.008   7.008   998.7715 < 2.2e-16 ***
cVar           1  86.149  86.149 12278.1766 < 2.2e-16 ***
rep            1   3.496   3.496   498.2492 < 2.2e-16 ***
Model:Gcor     2   0.648   0.324    46.1442 < 2.2e-16 ***
Model:Ecor     2   0.766   0.383    54.5668 < 2.2e-16 ***
Model:h2       2   0.976   0.488    69.5188 < 2.2e-16 ***
Model:cVar     2  11.205   5.603   798.5123 < 2.2e-16 ***
Model:rep      2   3.570   1.785   254.3959 < 2.2e-16 ***
Gcor:rep       1   0.059   0.059     8.3805 0.0037984 **
Ecor:rep       1   0.037   0.037     5.2937 0.0214165 *
h2:rep         1   0.316   0.316    44.9906 2.053e-11 ***
cVar:rep       1   0.075   0.075    10.6973 0.0010754 **
```

```
fraE:Gcor      1   0.010   0.010     1.4490 0.2287156
fraE:h2        1   0.266   0.266    37.9198 7.565e-10 ***
Gcor:Ecor      1   0.221   0.221    31.5360 1.994e-08 ***
Gcor:cVar      1   0.077   0.077    10.9774 0.0009245 ***
Ecor:h2        1   0.052   0.052     7.3897 0.0065676 **
Ecor:cVar      1   0.034   0.034     4.9045 0.0268024 *
h2:cVar        1   4.038   4.038   575.5661 < 2.2e-16 ***
Residuals  14550 102.089   0.007
---
Signif. codes:  0 ***,0.001 **,0.01 *,0.05 .
```

Table S4. ANOVA table of the final model after step-wise regression for data simulated with SD competition function. Correlation between true and predicted values of competition genotypic effect is the response variable. Explanatory variables are same as those described in Table S3.

```
Analysis of Variance Table

Response: COR
             Df Sum Sq Mean Sq   F value    Pr(>F)
Model         2 38.840 19.4202 4631.2226 < 2.2e-16 ***
fraE          1  0.010  0.0104    2.4906   0.11459
Gcor          1  0.460  0.4600  109.6875 < 2.2e-16 ***
Ecor          1  0.007  0.0074    1.7593   0.18477
h2            1  1.616  1.6158  385.3231 < 2.2e-16 ***
cVar          1 11.645 11.6452 2777.0841 < 2.2e-16 ***
Model:Gcor    2  0.161  0.0806   19.2226 4.825e-09 ***
Model:h2      2  0.200  0.1000   23.8466 4.925e-11 ***
Model:cVar    2  4.200  2.1002  500.8430 < 2.2e-16 ***
fraE:Ecor     1  0.013  0.0130    3.0921   0.07873 .
Ecor:cVar     1  0.018  0.0183    4.3743   0.03654 *
```

```
h2:cVar       1  0.753  0.7532  179.6171 < 2.2e-16 ***
Residuals  5023 21.063  0.0042
---
Signif. codes:  0 ***,0.001 **,0.01 *,0.05 .
```

Table S5. Best model selected compared to the base for traits root dry matter content (DM), root fresh yield (FYLD) and shoot weight (SHTWT) at harvest, and harvest index (HI) from various experiments. Only the values of base are given in scenarios where it is the best model. Number of observations and unique genotypes available for analysis is given below the trait. Other values are rounded to two decimal digits. Narrow sense heritability (h^2) of direct genotypic effect is given. Correlation is calculated based on the best linear unbiased prediction (BLUP) values and residual effects. Competition form is given bracketed with the maximum distance (Dist) covered and/or parameter values. Chi-square statistic, calculated from the log likelihood values of the Base and Model 1 or 2, is given with p-value (in brackets). Significant values at α = 0.1 are bolded.

**Data**	**Trait**	**Model**	**Variance**				**pRMSE**	**h²**	**Correlation**		**Competition Form**	**χ-square**
			σ_g^2	σ_c^2	σ_p^2	σ_r^2			ρ_{gc}	ρ_{cr}		
Ibadan_2013_C1	DM (511& 488)	Base	16.16	NA	NA	6.13	3.79	0.27	NA	NA	NA	1.44 (0.229)
		Model1	16.28	0.43	NA	5.56	3.79	0.28	0.01	-0.13	NN (along the long edge)	
	FYLD (646 & 617)	Base	7.43	NA	NA	19.79	4.96	0.68	NA	NA	NA	**9.31 (0.002)**
		Model1	7.15	0.35	NA	17.83	4.91	0.68	-0.05	0.00	Slow decay (Dist = 5m; *b* = 0.001)	
	HI (648 & 628)	Base	5e-3	NA	NA	9e-3	0.11	0.44	NA	NA	NA	**8.23 (0.004)**
		Model1	5e-3	< 5e-3	NA	8e-3	0.11	0.46	-0.05	-0.06	Fast decay (*k* = 0.4)	
		Base	10.20	NA	NA	11.89	4.19	0.57	NA	NA	NA	**15.52**

	SHTWT(660 & 631)	Model1	9.02	0.26	NA	10.76	4.12	0.59	-0.14	0.03	Slow decay (Dist = 5m; *b* = 0.001)	**(8.1e-05)**
Ibadan_2014_PYT	FYLD (152 & 81)	Base	40.54	NA	NA	77.01	10.6	0.63	NA	NA	NA	2.49 (0.114)
		Model1	35.87	2.829	NA	68.13	10.54	0.64	-0.15	-0.17	Slow decay (Dist = 10m; *b* = 0.001)	
	HI (154 & 81)	Base	6e-3	NA	NA	7e-3	0.11	0.46	NA	NA	NA	**3.17 (0.075)**
		Model1	6e-3	< 5e-3	NA	6e-3	0.11	0.40	-0.14	-0.08	Slow decay (Dist = 10m; *b* = 0.001)	
	*SHTWT(151 & 81)	Base	23.56	NA	NA	101.2	11.03	0.77	NA	NA	NA	**5.93 (0.01)**
		Model2	24.53	NA	27.46	60.52	10.53	0.67	-0.02	0.07	Competition error	
	DM(148 & 80)	Base	15.86	NA	NA	15.42	5.06	0.39	NA	NA	NA	NA
Ibadan_2014_C1	HI (282 & 265)	Base	0.02	NA	NA	< 5e-3	0.10	0.46	NA	NA	NA	**9.47 (0.002)**
		Model1	0.02	< 5e-3	NA	< 5e-3	0.10	0.46	0.05	-0.40	NN (all neighbors)	
	SHTWT (283 & 262)	Base	119.60	NA	NA	189.88	16.44	0.64	NA	NA	NA	NA
	DM(282 & 263)	Base	18.76	NA	NA	2.33	3.59	0.41	NA	NA	NA	NA
	FYLD (286 & 266)	Base	879.06	NA	NA	49.26	23.32	0.50	NA	NA	NA	NA
[illegible]		Base	25.10	NA	NA	4.39	4.10	0.32	NA	NA	NA	**3.24**

	DM (627 & 611)	Model1	25.71	0.16	NA	3.76	4.10	0.33	-0.05	0.06	Fast decay (α 1/Dist)	**(0.072)**
	SHTWT (781 & 753)	Base	13.31	NA	NA	17.19	4.99	0.65	NA	NA	NA	**4.95 (0.026)**
		Model1	13.66	0.17	NA	15.95	4.98	0.64	-0.16	-0.05	Slow decay (Dist = 5m; *b* = 0.001)	
	FYLD(758 & 735)	Base	8.61	NA	NA	24.00	0.18	0.70	NA	NA	NA	NA
	HI (757 & 736)	Base	0.007	NA	NA	0.01	0.13	0.51	NA	NA	NA	NA
Ikenne_2014_C1	DM (313 & 284)	Base	31.95	NA	NA	0.72	4.25	0.44	NA	NA	NA	**7.31 (0.006)**
		Model1	30.77	0.75	NA	< 5e-3	4.21	0.44	-0.06	-0.51	NN (along long edge only)	
	SHTWT(322 & 292)	Base	721.09	NA	NA	29.19	20.64	0.51	NA	NA	NA	NA
	FYLD(323 & 292)	Base	1037.0	NA	NA	30.61	25.01	0.51	NA	NA	NA	NA
	HI(321 & 292)	Base	0.04	NA	NA	<1e-3	0.14	0.99	NA	NA	NA	NA
Ikenne_2014_C2	SHTWT (367 & 342)	Base	32.53	NA	NA	37.81	7.81	0.58	NA	NA	NA	1.59 (0.207)
		Model1	31.97	0.65	NA	36.65	7.80	0.58	-0.02	-0.05	Fast decay (α 1/Dist)	

	FYLD(362 & 335)	Base	5.842	NA	NA	66.26	8.50	0.83	NA	NA	NA	NA
	DM(315 & 293)	Base	25.09	NA	NA	2.78	4.19	0.38	NA	NA	NA	NA
	HI(361 & 339)	Base	0.01	NA	NA	0.02	0.17	0.70	NA	NA	NA	NA
Mokwa_2013_C1	DM (571 & 537)	Base	15.98	NA	NA	8.86	4.10	0.37	NA	NA	NA	0.29 (0.588)
		Model1	15.99	0.01	NA	8.64	4.07	0.37	0.09	0.01	NN (all neighbors)	
	FYLD (734 & 694)	Base	2.47	NA	NA	18.87	4.60	0.84	NA	NA	NA	**3.69 (0.055)**
		Model1	2.27	0.38	NA	17.76	4.58	0.84	0.10	-0.01	Slow decay (Dist = 5m; *b* = 0.1)	
	HI (744 & 701)	Base	5e-3	NA	NA	9e-3	5.94	0.61	NA	NA	NA	**4.38 (0.036)**
		Model1	5e-3	< 5e-3	NA	9e-3	5.94	0.63	-0.01	-0.02	Fast decay (*k* =1)	
	SHTWT (749 & 707)	Base	3.98	NA	NA	32.03	5.94	0.74	NA	NA	NA	NA
Mokwa_2014_C1	DM (287 & 264)	Base	27.29	NA	NA	1.52	4.02	0.31	NA	NA	NA	1.60 (0.205)
		Model1	27.48	0.63	NA	1.27	4.02	0.31	-0.20	-0.22	Fast decay (α 1/Dist)	
	*FYLD (321 & 296)	Base	67.75	NA	NA	79.09	11.55	0.62	NA	NA	NA	**6.21 (0.01)**
		Model2	56.70	NA	17.38	53.52	11.35	0.65	0.07	0.12	Competition error	
	SHTWT(313 & 285)	Base	123.02	NA	NA	166.1	16.13	0.57	NA	NA	NA	NA

	HI(311 & 284)	Base	0.01	NA	NA	0.01	0.10	0.60	NA	NA	NA	NA
Mokwa_2014_C2	FYLD(311 & 286)	Base	3.61	NA	NA	16.91	4.47	0.73	NA	NA	NA	NA
	DM(260 & 239)	Base	13.95	NA	NA	9.89	4.32	0.36	NA	NA	NA	NA
	HI(310 & 286)	Base	0.09	NA	NA	0.01	0.13	0.61	NA	NA	NA	NA
	SHTWT(324 & 300)	Base	1.74	NA	NA	37.67	6.27	0.91	NA	NA	NA	NA

Table S6: Results from Model 5 (GS – spatial – competition model) compared to base GS model for traits that showed significant spatial and/or competition effect. Chi-square statistic is calculated from the log likelihood values of Base, or other models against Model 5 are given with p-value (in brackets) at α = 0.1 on their respective rows. For example, χ-square statistic in the row of Model 1 is the result of the comparison between Model 1 and Model 5. Significant values are bolded.

Data	Trait	Model	Variance					h^2	Spatial structure	Competition Form	χ-square
			σ_g^2	σ_c^2	σ_p^2	σ_s^2	σ_r^2				
Mowa_2013_C1	FYLD (734 & 694)	Base	2.47	NA	NA	NA	18.87	0.84	NA	NA	**7.47 (0.024)**
		Model1	2.27	0.38	NA	NA	17.76	0.84	NA	SD (Dist = 5m; *b* = 0.001)	**3.77 (0.052)**

		Model5	1.73	0.36	NA	1.03	17.997	0.85	Gaus – Range (φ = 4.5)	SD (Dist = 5m; *b* = 0.001)	
Ibadan_2014_C1	HI (282 & 265)	Base	0.02	NA	NA	NA	<5e-3	0.46	NA	NA	**10.62 (0.005)**
		Model1	0.02	<5e-3	NA	NA	<5e-3	0.46	NA	NNc (all neighbors)	1.15 (0.284)
		Model5	0.02	<5e-3	NA	1e-3	<5e-3	0.45	Gaus – Range (φ = 140)	NNc (all neighbors)	
Ikenne_2013_C1	DM (627 & 611)	Base	25.10	NA	NA	NA	4.39	0.32	NA	NA	**7.89 (0.019)**
		Model1	25.71	0.16	NA	NA	3.76	0.33	NA	FD (α 1/Dist)	**4.67 (0.031)**
		Model5	26.96	0.02	NA	0.84	2.90	0.32	Gaus – isotropic (φ = 9)	FD (α 1/Dist)	
Ibaadan_2014_PYT	SHT WT (151 & 81)	Base	23.56	NA	NA	NA	101.2	0.77	NA	NA	**16.15 (0.0003)**
		Model2	24.53	NA	27.46	NA	60.52	0.67	NA	Competition error	**10.21 (0.001)**
		Model5	27.78	NA	20.41	16.62	53.20	0.51	Sph – Range (φ = 0.5)	Competition error	

Figure S1: Original observation (column 1), direct genotypic BLUP (column 2), and competition BLUP OR competition error BLUP* (column 3) from model for A) dry root weight (DM), B) harvest index (HI), C) fresh root yield (FYLD), and D) fresh shoot weight (SHTWT). Depicted below are the trials/traits which exhibited significant genetic competition from model 1: A.1) IKN_2014_C1, A.2) IKN_2013_C1, B.1) IBA_2014_C1, B.2) IBA_2014_PYT, B.3) IBA_2013_C1, B.4) MOK_2013_C1, C.1) IBA_2013_C1, C.2) MOK_2013_C1, C.3) *MOK_2014_C1, and D.1) IKN_2013_C1. Missing values were linearly interpolated.

A.1. IKN_2014_C1 - DM

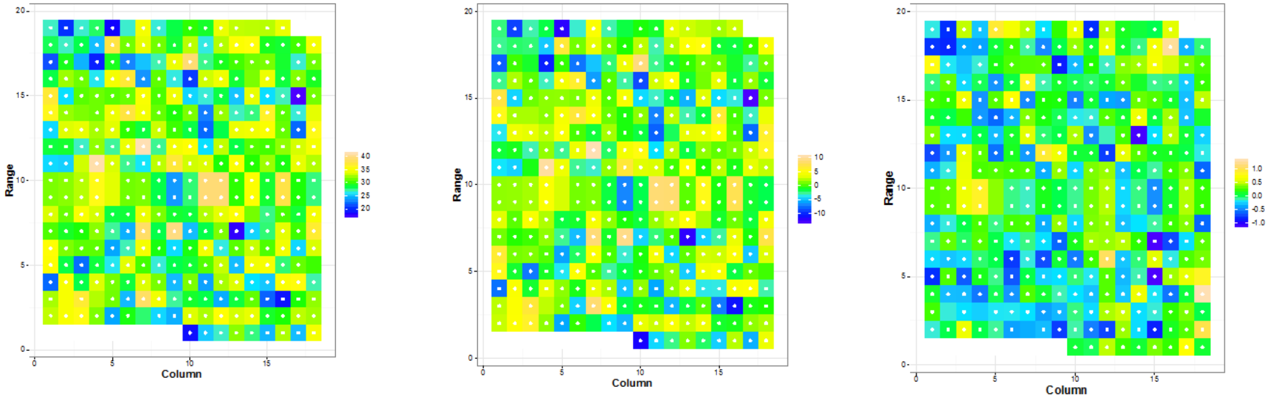

A.2. IKN_2013_C1 - DM

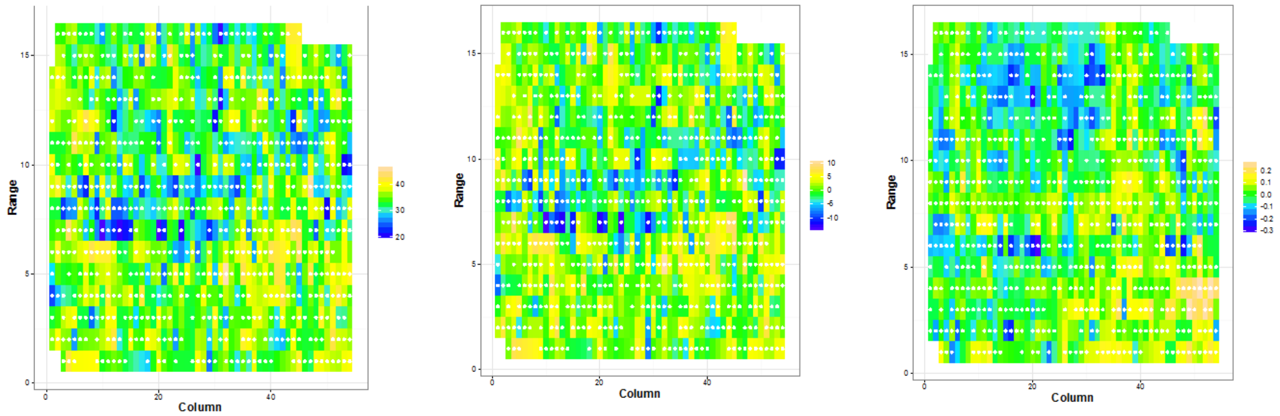

B.1. IBA_2014_C1 - HI

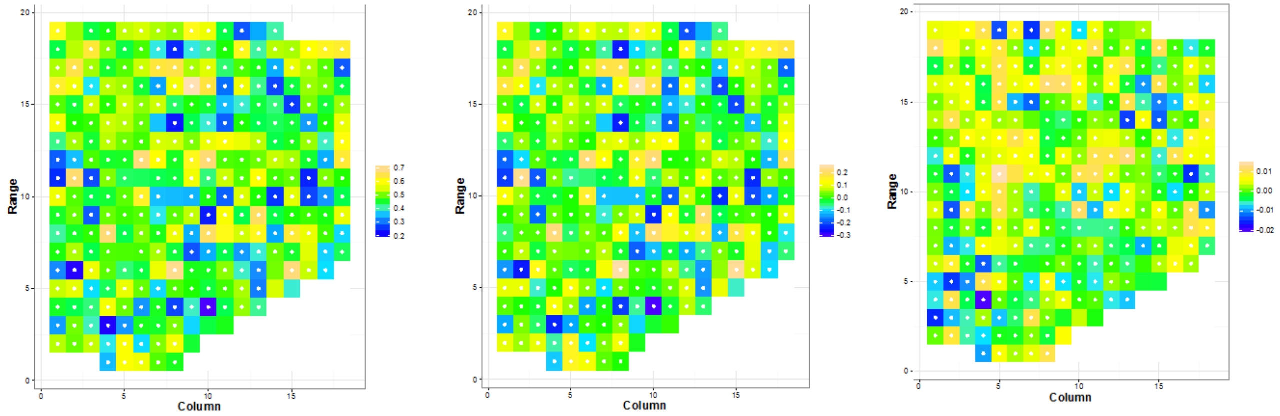

B.2. IBA_2014_PYT - HI

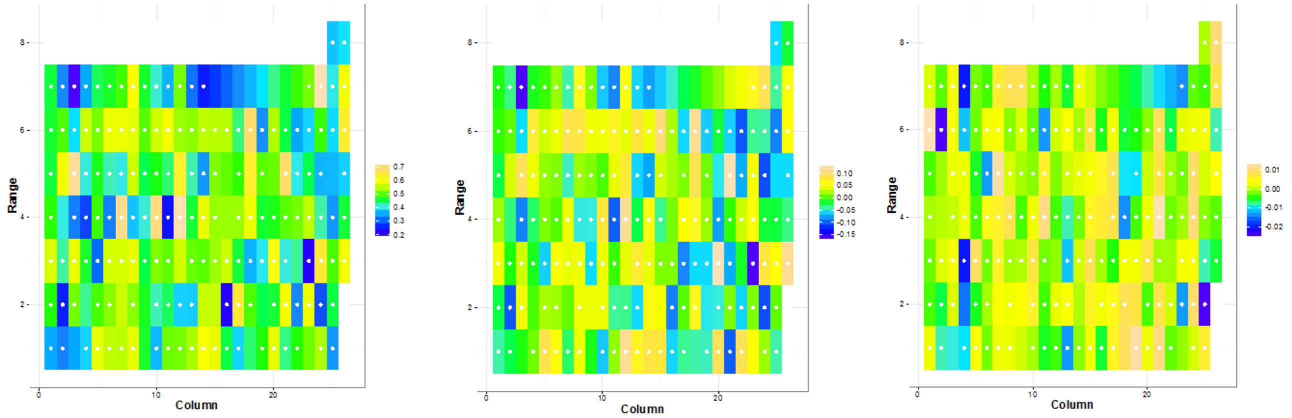

B.3. IBA_2013_C1 - HI

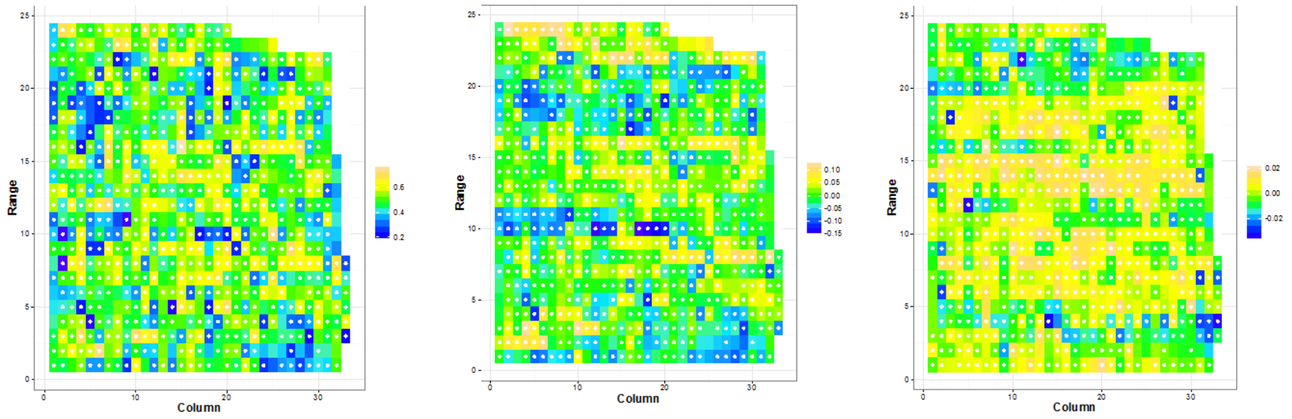

B.4. MOK_2013_C1 - HI

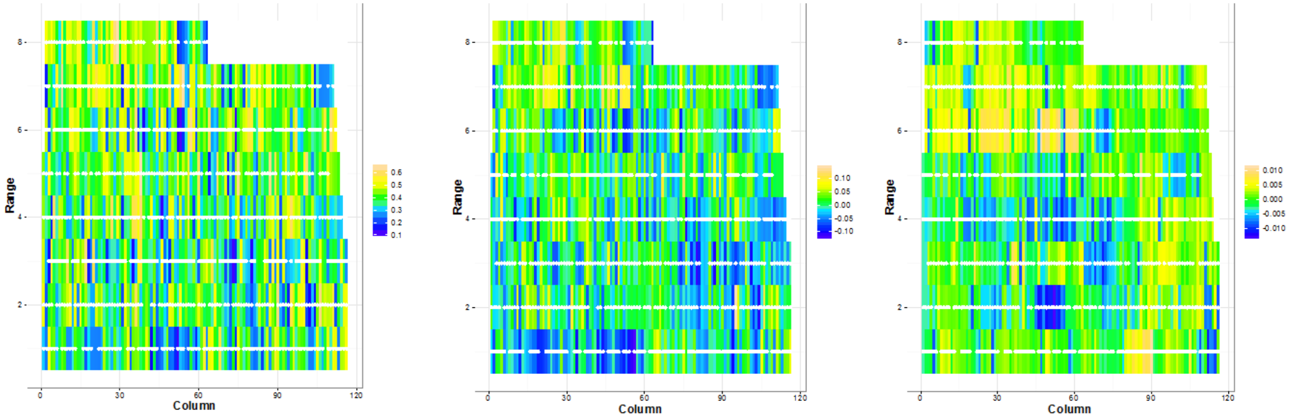

C.1. IBA_2013_C1 - FYLD

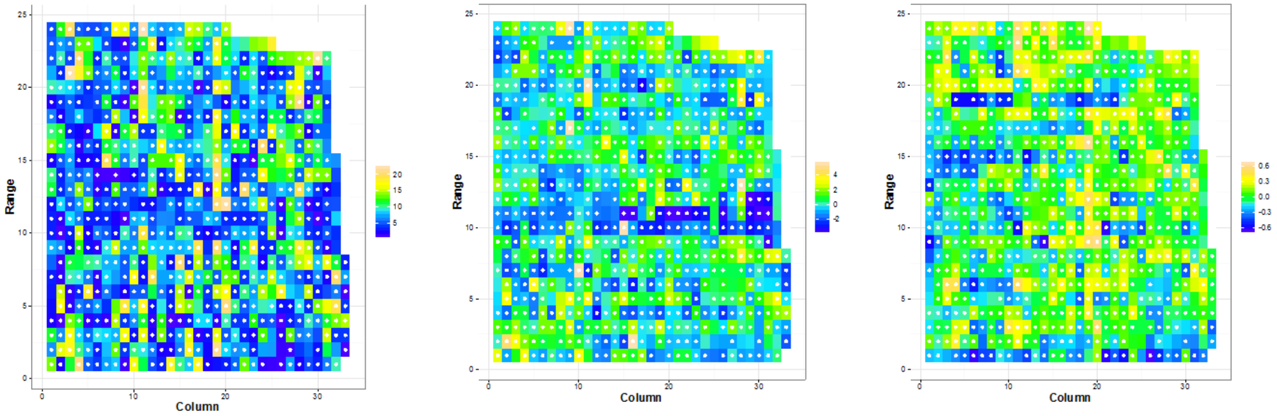

C.2. MOK_2013_C1 - FYLD

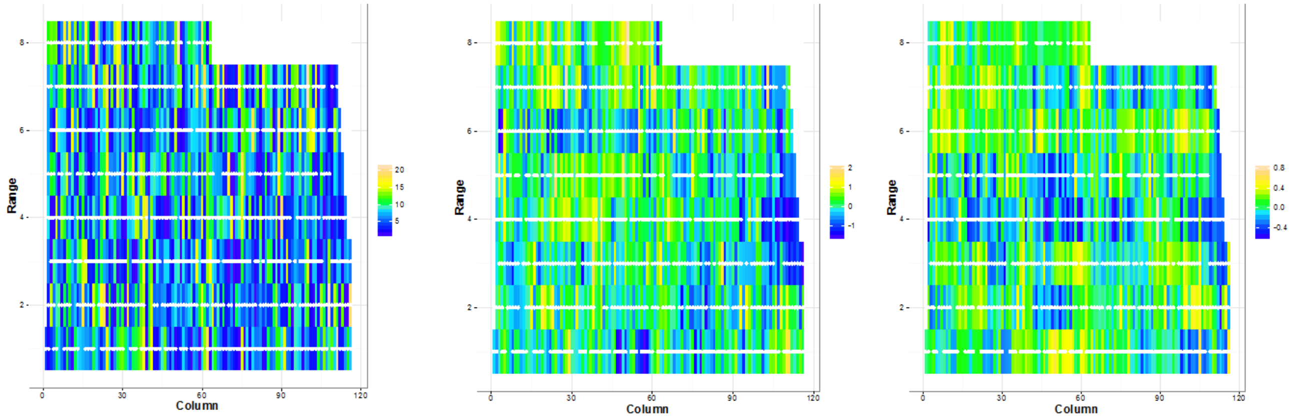

C.3.*MOK_2014_C1 - FYLD

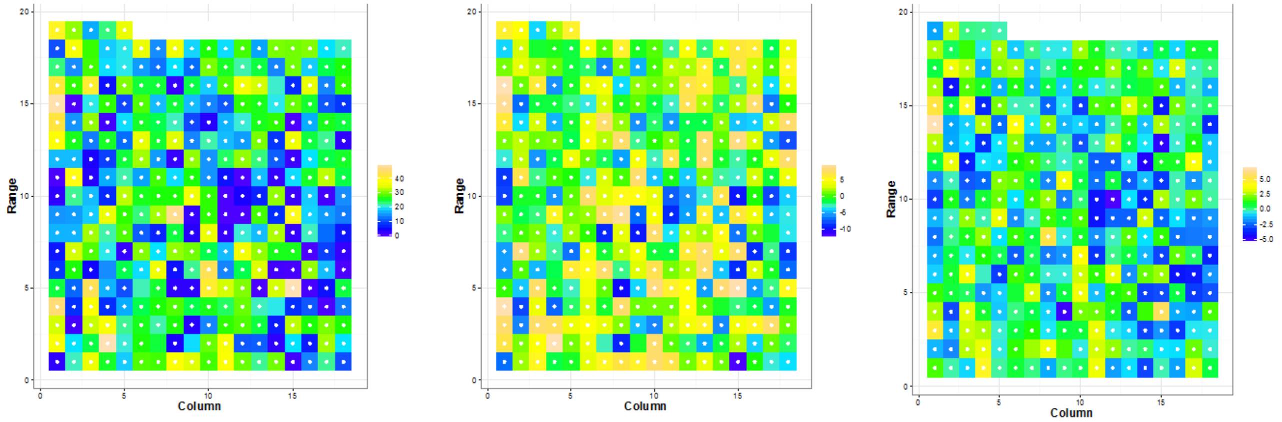

D.1. IKN_2013_C1 - SHTWT

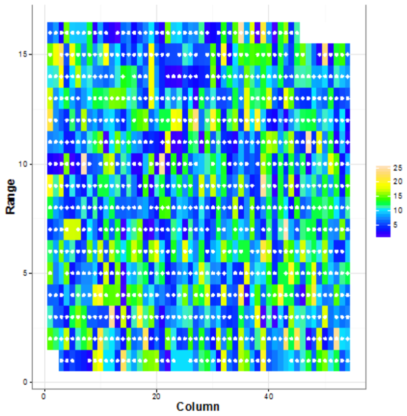

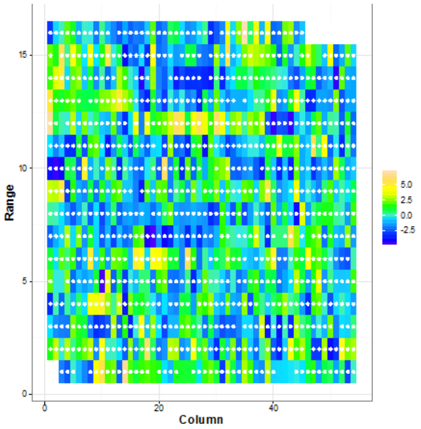

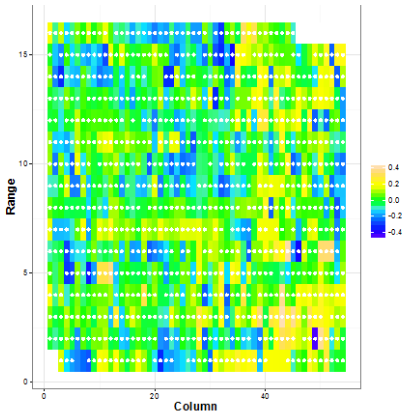

Supplement: Supplementary file 1 [file 933FileS1.pdf]
